# Meta-Omic Platforms to Assist in the Understanding of NAFLD Gut Microbiota Alterations: Tools and Applications

**DOI:** 10.3390/ijms15010684

**Published:** 2014-01-07

**Authors:** Federica Del Chierico, Daniela Gnani, Pamela Vernocchi, Andrea Petrucca, Anna Alisi, Bruno Dallapiccola, Valerio Nobili, Putignani Lorenza

**Affiliations:** 1 Unit of Parasitology, Bambino Gesù Children’s Hospital, IRCCS, Piazza Sant’Onofrio, 4, Rome 00165, Italy; E-Mails: federica.delchierico@opbg.net (F.D.C.); pamela.vernocchi@opbg.net (P.V.); andrea_petrucca@yahoo.com (A.P.); 2 Unit of Metagenomics, Bambino Gesù Children’s Hospital, IRCCS, Piazza Sant’Onofrio, 4, Rome 00165, Italy; 3 Liver Research Unit, Bambino Gesù Children’s Hospital, IRCCS, Piazza Sant’Onofrio, 4, Rome 00165, Italy; E-Mails: daniela.gnani@yahoo.it (D.G.); anna.alisi@opbg.net (A.A.); valerio.nobili@opbg.net (V.N.); 4 Interdepartmental Centre for Industrial Research-CIRI-AGRIFOOD, Alma Mater Studiorum, University of Bologna, Piazza Goidanich, 60, Cesena-FC 47521, Italy; 5 Department of Diagnostic Science, Sant’Andrea Hospital, Via di Grottarossa 1035, Rome 00185, Italy; 6 Scientific Directorate, Bambino Gesù Children’s Hospital, IRCCS, Piazza Sant’Onofrio, 4, Rome 00165, Italy; E-Mail: bruno.dallapiccola@opbg.net; 7 Hepato-Metabolic Disease Unit, Bambino Gesù Children’s Hospital, IRCCS, Piazza Sant’Onofrio, 4, Rome 00165, Italy

**Keywords:** pediatric patients, non-alcoholic fatty liver disease, non-alcoholic steatohepatitis, obesity, metabolic syndrome, systems biology, gut microbiota, diet, meta-omic platforms, data integration

## Abstract

Non-alcoholic fatty liver disease (NAFLD) is the most common cause of chronic liver disease worldwide as a result of the increasing prevalence of obesity, starting from early life stages. It is characterized by a spectrum of liver diseases ranging from simple fatty liver (NAFL) to steatohepatitis (NASH), with a possible progression to fibrosis, thus increasing liver-related morbidity and mortality. NAFLD development is driven by the co-action of several risk factors, including obesity and metabolic syndrome, which may be both genetically induced and diet-related. Recently, particular attention has been paid to the gut-liver axis, which may play a physio-pathological role in the onset and progression of the disease. The gut microbiota is intended to act as a bioreactor that can guarantee autonomous metabolic and immunological functions and that can drive functional strategies within the environment of the body in response to external stimuli. The complexity of the gut microbiota suggests that it behaves as an organ. Therefore, the concept of the gut-liver axis must be complemented with the gut-microbiota-liver network due to the high intricacy of the microbiota components and metabolic activities; these activities form the active diet-driven power plant of the host. Such complexity can only be revealed using systems biology, which can integrate clinical phenomics and gut microbiota data.

## 1. Introduction

In the past decade, the incidence of non-alcoholic fatty liver disease (NAFLD) has reached epidemic proportions, and NAFLD has become one of the most frequent causes of chronic liver disease worldwide [1,2]. The term NAFLD covers various liver abnormalities that are observable during a liver biopsy: an intrahepatic accumulation of fat alone (NAFL) or in association with various degrees of necrotic inflammation and possibly fibrosis (non-alcoholic steatohepatitis, NASH) [1–5]. It has been reported that NAFLD in susceptible individuals can evolve to cirrhosis and hepatocellular carcinoma, which eventually require liver transplantation [6]. Recent advances demonstrate that NAFLD is a complex multifactorial disease; in fact, it requires the coexistence of multiple factors, including host (gene polymorphisms) and environmental factors as well as those related to lifestyle (*i.e*., dietary habits) and behavior. Gene polymorphisms affect the metabolism of dietary compounds, which in turn influence the expression of the proteins involved in several important cellular processes, such as the response to oxidative stress, inflammation and the innate immune response [5,7,8]. Together, these factors create a network of interactions that lead to NAFL and progress to NASH [9–11]. The pathophysiological mechanisms leading to the development and progression of NAFL remain unknown [11,12]. It has been hypothesized that NAFL can be caused by the increased delivery of free fatty acids (FFA) to the liver, the disordered metabolism of fatty acids (FA) by hepatocytes, or the increased *de novo* synthesis of FA and triglycerides [13]. Several elements can cooperate in promoting intrahepatic FFA accumulation and insulin resistance, including genetic and epigenetic regulations; external stimuli such as high caloric and high-fat diet (HFD) and/or high-sugar foods, combined to low physical activity [14–16].

However, NAFL development may not just be a consequence of hepatogenous stimuli. In fact, tissues and organs, such as adipose tissue and the gut, which are influenced by genetics and diet, could induce secondary effects, including the overproduction and release of pro-inflammatory adipocytokines, an increase in oxidative stress, and the activation of the innate immune response [14–17]. The elevated susceptibility of a fatty liver to these factors might also explain the progression to NASH and the activation of the molecular mechanisms that lead to both liver fibrosis and the severe multi-organ damage that characterizes cardiometabolic syndrome [3,18–23].

In addition to steatosis, hepatocellular degeneration (ballooning, Mallory hyaline), mixed inflammatory cell infiltration and eventually fibrosis are the critical features of NASH. Although the relationship between steatosis and metabolic factors (e.g., overnutrition, insulin resistance, hyperglycemia, metabolic syndrome, and hypoadiponectinemia) has mostly been described, less is known about the inflammation recruitment during the lipotoxicity of hepatocytes despite its crucial importance for the perpetuation of liver injury and fibrogenesis. In fact, it has been reported that injured hepatocytes may play a crucial role in the induction of the low-grade inflammatory state that characterizes NASH: the injured hepatocytes may release molecules containing damage-associated molecular patterns (DAMPS). In addition to DAMPS, molecules containing pathogen-associated molecular patterns (PAMPs) are also derived from the gut. Lipopolysaccharides (LPSs), one type of PAMP, are up-regulated in the circulatory system of NASH patients affected by necro-inflammation and severe liver damage [24]. PAMPs and DAMPs may activate Toll-like receptor-4 (TLR-4) signaling, resulting in the gene expression of pro-inflammatory cytokines, including tumor necrosis factor (TNF)-α and several interleukins (ILs) that may exacerbate hepatocellular damage [25]. Lastly, the gut microbiota may contribute to hepatic fibrogenesis by activating hepatic stellate cells (HSCs) via a direct LPS/TLR pathway in these cells or via TLR-dependent recognition of certain bacteria products by Kupffer cells (KCs) and hepatocytes in the liver [24–26]. In addition, the gut microbiota alters nutrient absorption, energy homeostasis and intestinal permeability; the latter promotes the translocation of bacteria-derived products from the intestinal lumen to the circulation and causes a systemic inflammatory state that contributes to the metabolic derangement that occurs during NAFLD pathogenesis [27]. Hepatocellular inflammation may be secondary to the altered intestinal permeability and translocation of either intact bacteria or microbial products to the circulation, which in turn reach the liver and are correlated with NAFLD [28–30].

The gut flora is perturbed in a large percentage (20%–75%) of patients with chronic liver diseases, and metabolic diseases are the product of multiple triggering factors. Therefore, modulation of the gut microbiota may be a new method of treating or preventing NAFLD [31,32]. Indeed, the bacterial metabolism of carbohydrates and proteins increases short chain fatty acids (SCFA) and a range of other metabolites, including those from aromatic amino acid (AAA) fermentation. In addition, fiber-related molecules, such as polyphenols, are produced by microbial catabolism, and aromatic moieties are released into the colon. Moreover, the quaternary ammonium salt choline is metabolized by bacterial reactions, providing trimethylamine (TMA) from dietary choline in a reaction catalyzed by enzymes within the gut microbiota [33].

Furthermore, the inflammation and damage in NASH could also depend on foreign gut microbiota products, which in turn could promote the release of pro-inflammatory and/or pro-fibrogenic molecules by activating the TLR pathway in KCs and HSCs [34]. NAFLD pathogenesis remains incompletely understood; however, a potential role of the gut microbiota in obesity has recently been established. Specific gut microbiota patterns may be associated with changes in energy harvesting and storage in a mouse model [23]. Subsequently, increased energy extraction has been associated with obesity, which has been shown to be a trait that is transmissible by microbiota colonization [35]. Furthermore, the mucosal permeability and translocation of bioactive gut molecules tightly regulate the low-grade inflammation response due to the balance between tolerance and the immune response; then, the imbalance leads to most of the complications in the nearby and distant organs [36,37]. In particular, the gut microbiota modulates dietary choline and bile acid metabolism, and it is an endogenous source of ethanol [37]. Deregulation of this complex network of interactions can lead to the onset of NAFLD or even drive the progression of NAFLD towards NASH [38].

Due to the complexity of the disease, standard therapeutic strategies, which are based on weight loss and lifestyle changes, are often unsuccessful for treating NAFLD [1]. Treatments directed against insulin resistance and oxidative stress have also been proven to be unsatisfactory [1]. Therefore, innovative pharmacological tools must be developed. Among the new strategies recently proposed, the use of prebiotics and probiotics should be considered; these compounds play a role in modulating diet-altered and inflammatory gut microbiota. Although their potential effects on the NAFLD condition have recently been investigated in animal models and in some human studies, additional randomized controlled clinical trials are needed to demonstrate the efficacy of these dietary components in the treatment of NAFLD [39]. However, it is reasonable to assume that a combined pharmacological approach that targets lifestyle habits and both novel and old patho-mechanisms (e.g., insulin resistance, oxidative stress, gut-liver axis, and apoptosis) could be the best option for NAFLD therapy [40,41].

**Figure 1.**
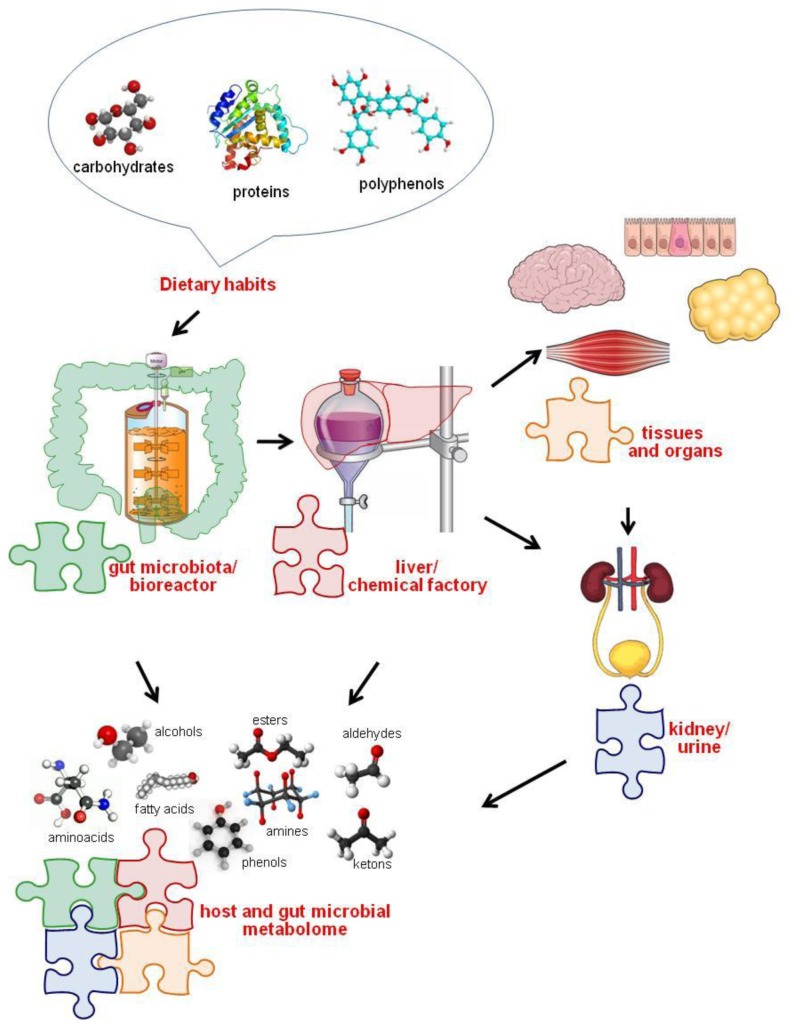
The “bioreactor” colon model. Diet triggers “bioreactor”-colon and “factory”-liver reactions through a concerted action, leading to single chemical components. These components form the entire combined host and gut microbial metabolome.

This review will focus on the gate-keeper function of the intestines, which actively complements the commensal host metabolic activities via the gut-liver axis (Figure 1). This paper provides an overview of current evidence for the role of the gut microbiome in NAFLD pathogenesis and discusses a “deciphering” code to reveal the role of the gut microbiota within the gut-microbiota-liver axis using an approach with multiple levels of complexity: (i) a description of the gut microbial communities by exploiting both genomics- and metagenomics-based strategies (descriptive level, enterotype); (ii) elucidation of the gut microbial metabolic activities and evaluation of how microbial networks alter the entire metabolism using metabolomics approaches (functional level, metabotype); and (iii) metabonomic analyses of the contribution of diet, which is intended to be an external stimulus, to the microbial and host metabolic network and to the gut homeostasis (nutritional level, exposome). These different levels of complexity can only be unveiled by systems biology, which can integrate NAFLD clinical phenomics and large data sets from the gut microbiota. The physicochemical modifications of the “bioreactor” intestine can suggest targeted strategies for NAFLD intervention that are directed towards controlling and modifying the function of gut microbiota; this control may be achieved through diet-linked solutions.

## 2. The New “Omics” Era and the Understanding of the Gut Microbiota in NAFLD: Descriptive and Functional Meta-Omics Approaches

Over the past five years, a large number of studies have been performed to highlight the relationship between gut microbiota and health maintenance. This effort has employed the new concept of a coupled host and microbial metabolism that is associated with the “core” microbiome. Recently, “omics” scientists have started to extend beyond the descriptive level and investigated a more advanced functional level of the gut microbiota; this type of approach is becoming essential for investigating diseases such as NAFLD/NASH, which are closely related to diet and metabolic alterations.

**Figure 2.**
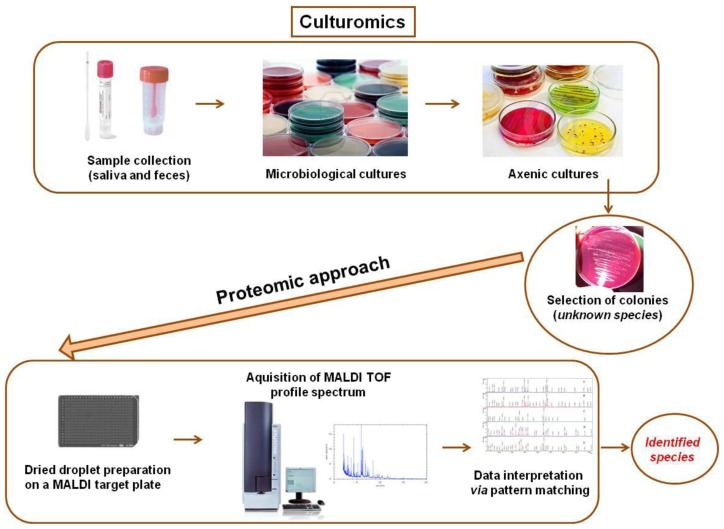
Culturomics-based investigations of gut microbiota. Scheme of pre-analytical and analytical steps of the matrix-assisted laser desorption/ionization time-of-flight (MALDI-TOF)-based approach for culturomics investigations. Samples from the gut or other microbiota (e.g., feces and saliva) are assayed on solid media selective for axenic cultivation. Isolated microbial colonies are subjected to peptide extraction before MALDI-TOF MS processing and species identification (ID) by peptide fingerprinting.

Of the thousands of microbe species inhabiting the intestine, few of them are actually cultivable. However, culturomics still plays a crucial role in the full description of the gut microbiota, especially when the microbiota is characterized by unusual and atypical operational taxonomic units (OTUs) [42] and when the operational pipeline includes large reference databases of the peptide fingerprinting obtained from matrix-assisted laser desorption/ionization time-of-flight mass spectrometry MALDI-TOF MS-based proteomics [43] (Figure 2). Many antibiotic regimens reduce both the richness and the abundance of the gut microbiota. Dubourg *et al*. [42] analyzed stools collected from a patient treated for drug-resistant *Mycobacterium tuberculosis*. The authors performed cultures in 70 conditions and identified the results using MALDI-TOF MS and 16S rRNA-based sequencing and pyrosequencing. Only 39 bacterial species were identified using the culture, including one new species and three species that had not been previously observed in the human gut microbiota. Interestingly, a next generation sequencing (NGS) approach showed only 18 phylotypes; thus, NGS detected a smaller number of bacterial species than the culture-based analysis. Only two phylotypes were shared with the culturomics. The authors recovered more cultivable than non-cultivable bacterial species, possibly because of the low bacterial loading in the gut microbiota; these results thus demonstrated the depth bias of NGS. In another study, culturomics allowed the isolation of 31 new bacterial species, giant bacteria and Archaea; this study also detected the greatest number of large human viruses [44]. Interestingly, using culturomics, the same group [45] studied the gut microbiota of two lean Africans and one obese European. Their study used 212 different culture conditions and combined MALDI-TOF MS technology to investigate both NGS and Sanger sequencing. The culturomics-based approach provided 32,500 different morphotypes associated to 340 bacterial species, including two from the rare Deinococcus-Thermus and Synergistetes phyla and 174 associated to the typical human gut.

The NGS analysis produced 698 phylotypes, including 282 known species, only 51 of which overlapped with the culturomics-based microbiota.

The advent of high-performance platforms based on molecular techniques has surely opened new avenues other than culturomics for obtaining deep knowledge about the gut microbiota components. These new technological tools have further highlighted the complexity of the microbiota ecosystem and extended the identification to new bacterial populations that were not yet characterized, thus freeing scientists from the old culture-dependent approach [46]. The most important techniques utilize the following: (i) oligonucleotide probes and oligonucleotides that hybridize sequences of ribosomal RNA on platforms such as DNA microarrays and fluorescent *in situ* hybridization (FISH); these techniques provide a discrete genomics approach, and their species representativeness has progressively improved [47]; (ii) heterogeneity of PCR profiles of complex communities obtained by denaturating and temperature gradient electrophoresis (e.g., PCR-DGGE and PCR-TGGE) [48]; (iii) real-time PCR for both qualitative and quantitative analysis; and (iv) metagenomics using NGS platforms characterized by different chemistry, technological platforms and bioinformatic processing workflows [49].

**Figure 3.**
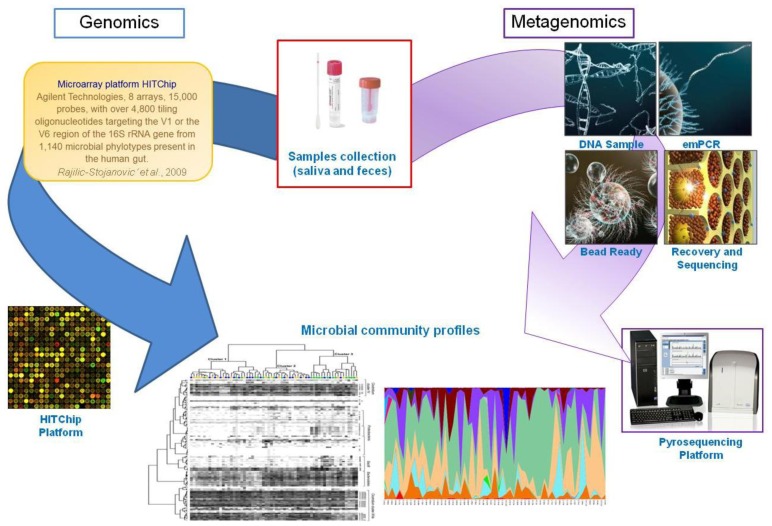
Genomics- and metagenomics-based investigations of gut microbiota. After standardized DNA extraction and quality control (QC) protocols, which are included in the workflow for assaying DNA purity and concentration, metagenomic sequences from the gut or other microbiota (e.g., feces and saliva) are generated by pyrosequencing selected 16S rRNA regions from microbial genomes.

Currently, genomic- and NGS-based technologies can overcome many of the culture-based limiting issues and thus radically improve the understanding of the host patho-physiological modifications related to gut microbiota modifications (Figure 3). However, bias in the OTU description, discovery of new OTUs and OTU cataloguing must always be considered because of the constraints in measuring the relative abundance; these constraints are caused by specific biological traits of the “systems”, DNA extraction procedures [50], probe design and available databases [51] even during the new NGS “era” and require a constant parallel culturomics pipeline for the phylotypes description. Hence, the study of the entire microbiota metagenome has been started at the population level [52].

The bacterial species composition varies among individuals and over time in the same individual, but the activities encoded by the microbiome appear to be more stable. This stability is not surprising because the majority of the microbial population shares a minimum set of genes that are required for adaptation to the intestinal environment. Therefore, studying both the diversity and species composition, as well as the metabolic characteristics, provides a valuable background for fully understanding healthy and diseased states. Recently, metagenomic studies of mucosal and fecal samples obtained from healthy subjects have shown the presence of Firmicutes, Bacteroidetes, Proteobacteria, Fusobacterium, Verrucomicrobia, Cyanobacteria, and Actinobacteria Spirochaetes in large populations [53–57]. However, it is difficult to find Bifidobacteria species in metagenomic libraries, although these species are among the most abundant species when identified by traditional microbiological methods [53,58,59]. In addition to metagenomics, metabolomics is also currently used to analyze microbiota and study metabolic organization. While genome-wide association studies have found associations between disease genotype and phenotype changes, metabolome-wide association studies have correlated the metabolic phenotypes with the disease phenotypes [60]. Through the production of antimicrobial compounds, volatile fatty acids and chemically modified bile acids, the gut microbiota creates a very metabolically reactive environment, which is often described as a bioreactor [61,62]. Recent studies of fecal extracts have shown that metabolic analyses using proton nuclear magnetic spectroscopy (^1^H-NMR) and gas chromatography-mass spectrometry (GC-MS) can clarify the interspecies metabolic differences of microbiota components [63], thus providing important diagnostic information about the main intestinal diseases [51]. Metabolomics acts as tool to contextualize metabolic profiles into a systems biology framework and, in the case of “quantitative measure of the metabolic response of living systems to patho-physiological stimuli or genetic modification”, respond to the definition of metabonomics [64]. In accordance with the structural components of their cells, the gut microbiota communicates with the host, which has a characteristic secretion profile; thus, the microbiota participates in the host metabolism. This secretome or metabolome of small molecules can be detected in both feces and urine [65]. Advances in ^1^H-NMR, GC-MS and liquid chromatography mass spectrometry (LC-MS) technologies enable monitoring of the concentration and chemical property changes in the metabolites. Combining metabolic profiles with multivariate analyses now constitutes a new approach for examining the metabolic host-microbiota cooperation for various types of phenotypes, pathologies and diets [66]. In particular, the combined analysis of the metabolome in various biological fluids, including the extracts of fecal water, plasma and urine, is a viable strategy for determining the connections between the bioconversion of non-digestible food ingredients, their bio-availability and their effect on the host metabolism; these connections can also be related to the current diseases [67].

**Figure 4.**
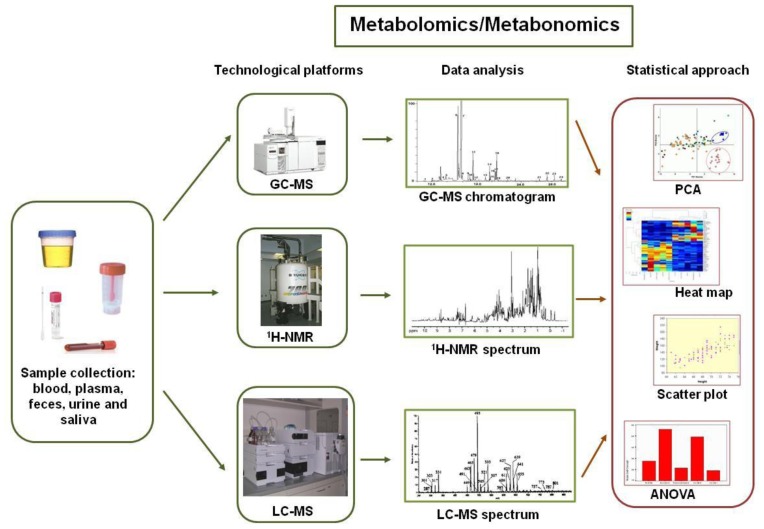
Metabolomics- and metabonomics-based investigations of gut microbiota. Samples, such as feces, urine, blood, plasma and saliva, can be studied using metabolomic approaches to detect metabolites, which are further analyzed within an integrated biocomputing framework. The metabolomic platforms include gas-chromatography mass spectrometry (GC-MS), proton nuclear magnetic spectroscopy (^1^H-NMR) and liquid chromatography mass spectrometry (LC-MS). The ^1^H-NMR and MS profiles are integrated, and the data are explored by multivariate statistical analyses (e.g., PCA, heat maps, scatter plots, and ANOVA) to describe the pathways of both the microbial and host metabolism and to correlate alterations in these pathways with disease-related phenotypes.

Therefore, besides genomics and metagenomics, metabolomics and metabonomics (Figure 4) allow for the analysis of catabolic metabolites and food-linked xenobiotics (foodomics), both molecular classes particularly important in the digestive chemical pathways of the “bioreactor” gut.

**Figure 5.**
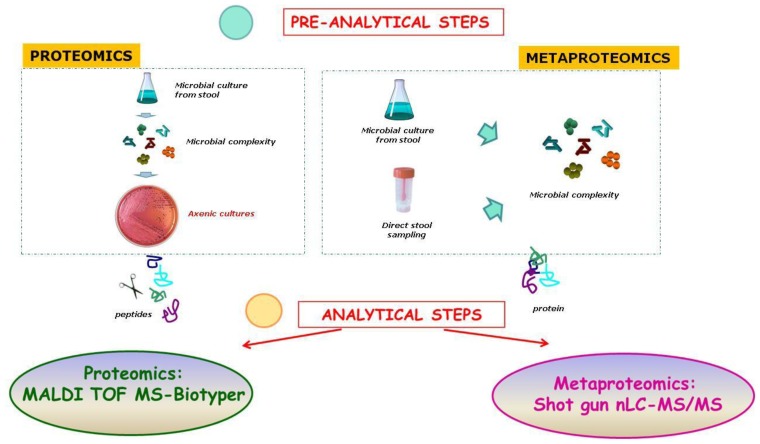
Proteomics- and metaproteomics-based investigations of gut microbiota. Microbial axenic cultures or direct stool samples are analyzed by MALDI-TOF MS or LC-MS/MS, providing fingerprinting profiles associated with the peptidome (proteomics) or the proteome (proteomics and metaproteomics). The two complementary analyses can be used to identify the OTU catalog of the gut microbiota. The full description is achieved through operational workflows that identify peptide OTUs or peptide protein OTUs.

However, investigation on gut microbiota protein reservoirs, categorized for functional groups (COGs), has recently received an important technical and conceptual advance, especially for two reasons: (i) the automatization of the bioinformatic pipelines linking identified peptides to bacterial OTUs through advanced metaproteomics workflows; and (ii) the differential processing of fecal contained- and mucosa adherent- bacteria [68]. Even with these advances, the complementary role of the proteomics and metaproteomics approaches must be critically considered (Figure 5).

Computational biology and statistical bioinformatics have become crucial for processing all of these heterogeneous data. Pyrosequencing raw reads are preliminarily analyzed by QIIME software [69] to guarantee a high level of accuracy in OTU detection by using a quality assay control (e.g., average quality score ≤25, read length and base calls). Sequences that pass the quality control filter are denoised [70], and singletons are excluded. Generally, OTUs with a 97% similarity are picked using the uclust method [71], and the representative sequences are submitted to the RDPII classifier [72] for taxonomy assignment and to the Greengenes 16S rRNA gene database [73] to determine the relative abundance of each OTU. The α-diversity can be computed by QIIME to produce rarefaction curves, Chao1 richness [74] and Shannon diversity indices [75], while UniFrac may evaluate the β diversity sequence trait [76].

**Figure 6.**
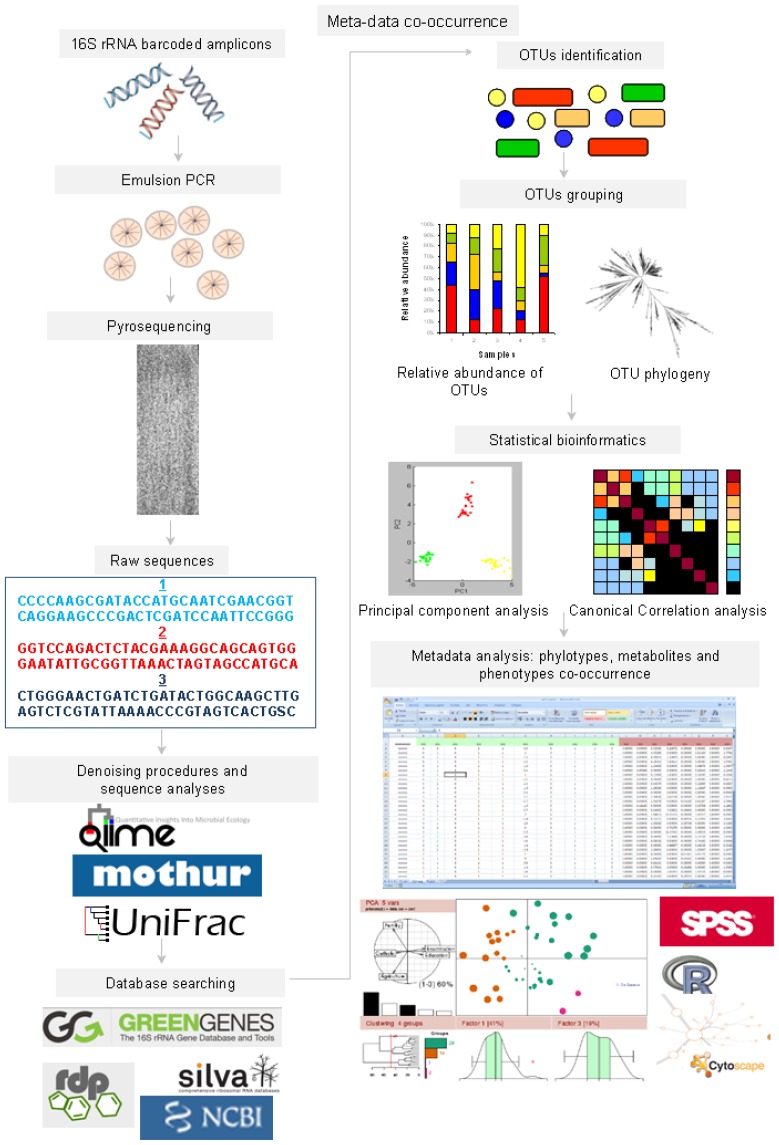
Bioinformatics pipelines for meta-omics data integration from gut microbiota data.

Weighted UniFrac distance matrices and OTU tables can be then related to each other in co-correlation plots by statistical tests (e.g., ANOVA, Adonis and Anosim) to verify the influence of discrete and continuous variable on the microbial population distribution. The OTU taxonomy table and the weighted UniFrac distance matrices, which are generated by QIIME [69], are used to produce heat maps for the OTUs and metabolites (*i.e*., their relative amounts compared with an internal standard); the heat maps are generally created using R software. Metabolic networks can be visualized by Cytoscape 2.5.2 [77]. Clearly, the design and application of dedicated bioinformatics pipelines, which can reduce calculation times and improve computing power, is now a priority to extend pilot studies on gut microbiota to clinically significant populations (Figure 6).

## 3. Diet-Gut Microbiota Interactions

### The Role of Diet in Shaping and Modulating the Gut Microbiota

Diet is one of the most important determinants of the microbial diversity of the gastrointestinal (GI) tract, and dietary components have the potential to affect microbial populations and their related distributions (*i.e.*, produce different individual enterotypes) beginning at the early stages of life [35,78–80]. The selected bacterial populations can, in turn, influence the physiological performance of the human host; this influence has been demonstrated between breastfeeding and either Lactobacilli [81,82] or enrichment of the entire bacterial community [83] in the newborn gut microbiota. After weaning, eating habits may influence the gut prevalence of microbiota phylotypes, as established by a plethora of studies showing the plasticity of the gut microbiota and its responsiveness to environmental factors. Generally, dietary changes appear to explain 57% of the total structural variation in gut microbiota, whereas genetic changes account for no more than 12% [84]. Therefore, diet may have a leading role in modulating the gut microbiota; diet may induce a switch in key phylotypes, which may subsequently have the potential to transform a healthy gut microbiota into a dysbiotic disease-inducing organ [85]. For example, the “Western” diet, which is rich in sugars and fats, seems to cause dysbiosis, which affects both the host GI metabolism and the immune homeostasis; this influence was demonstrated in a mouse model, in which human fecal microbiota was transplanted into germ-free mice [86]. In mice that were first fed a low-fat and then a sugar/fat rich diet, the microbiota shifted to become dominated by Firmicutes with a reduction in *Bacteroidetes* [86]. In other mice experiments, a carbohydrate-reduced diet induced overgrowth of Bacteroidetes phyla [87], while calorie-limited diets inhibited the growth of *Clostridium coccoides*, *Lactobacillus* spp. and *Bifidobacteria* spp., which are all major butyrate producers that are required for colonocyte homeostasis [88]. Complex carbohydrates increased the levels of beneficial *Bifidobacteria* spp. (e.g., *Bifidobacterium longum*, *Bifidobacterium breve* and *Bifidobacterium thetaiotaomicron*) [89]. Refined sugars, in contrast, induced overgrowth of *Clostridium difficile* with a subsequent increase in bile [90]. The large amount of fiber in a vegetarian diet appears to increase SCFA production and thus decrease the intestinal pH, preventing the growth of *E. coli* and Enterobacteriaceae in general [91]. Remarkably, European children have been found to have a microbiota that has lower amounts of Bacteroidetes and higher amounts of Enterobacteriaceae than those of rural African children; the authors attribute this difference to the low dietary fiber intake by Europeans [92]. Therefore, in addition to enterotypes [52], these data corroborate the hypothesis that gut microbiota phylotypes may vary geographically with modulations that respond to the host diet. In support of this hypothesis, a study on populations of *Bacteroides plebeius* has shown that this species is able to adapt to the local diet in different groups of people. In particular, Japanese strains of *B. plebeius* contain a gene that is acquired from marine bacteria and is required to degrade the polysaccharide porphyrin. This carbohydrate is found in edible seaweed, and the gene required for its digestion apparently does not occur in the microbiota strains of North American populations [93]. Therefore, it is highly probable that microbiota species adapt themselves to dietary changes through gene rearrangements and that, surprisingly, this adaptation is the result of gene exchanges with environmental bacteria [43].

In the past five years, the effect of the gut microbiota and the development and progression of NAFLD have been extensively studied not only in animal models but also in humans [26,94–101]. The intestinal microbiota has the potential to increase intra-hepatic fat through several mechanisms, such as altered appetite signaling, increased energy extraction from the diet, altered expression of genes involved in *de novo* lipogenesis or peroxidation, or inflammation-driven steatosis [28,29,34].

## 4. Gut Microbiota and Development of NAFLD

### 4.1. The Contribution of the Mouse Model

In addition to pivotal studies using mice [35,101], other remarkable papers have provided fundamental information on the modulation of the gut microbiota under external stimuli. Bull-Otterson *et al*. [102] fed mice with a liquid Lieber-DeCarli HFD supplemented with 5% *v/v* alcohol for 6 weeks and compared this diet to an alcohol-free HFD. Additionally, the probiotic *Lactobacillus rhamnosus* GG (LGG) was administered to a mice subgroup from six to eight weeks, and the intestinal permeability, hepatic steatosis (HS), inflammation and injury parameters of the animals were monitored (Table 1). Metagenomic analyses of the gut microbiota were performed by pyrosequencing the 16S rRNA V3–V5 regions. Prolonged ethanol-added feeding caused a decrease in the abundance of both Bacteroidetes and Firmicutes phyla with an increase in Proteobacteria and Actinobacteria phyla; the bacterial genera that showed the highest increase were *Alcaligenes* spp. and *Corynebacterium* spp. Combined with gut microbiota alterations, alcohol indeed caused an increase in plasma endotoxins, fecal pH, and hepatic inflammation. Interestingly, when LGG was administered, the ethanol-induced damage at the gut and liver level was inhibited, and the Firmicutes and Proteobacteria quantities were brought back to the control group levels [102].

HFD feeding is recognized to be a valid and widely applicable approach for studying the development and progression of obesity and metabolic disorders; this type of feeding has demonstrated that metabolic models can actually overcome genetic ones (e.g., gene knock-out). However, Le Roy *et al.* [99] have shown the role of the gut microbiota in NAFLD development using transplantation experiments in mice of specific genetic backgrounds. Interestingly, in this study, the authors demonstrated that some C57BL/6J mice fed a HFD developed hyperglycemia, systemic inflammation and steatosis; these mice were termed “responders.” In contrast, other mice did not display any significant metabolic change and were termed “non responders.” In the same study, microbiota transplant explants from the HFD-fed mice to the germ-free mice were performed to determine whether the microbiota may have a role in transmitting the “responder” or the “non-responder” phenotype. The researchers found some important differences between the two receiver groups. After 16 weeks, the two groups showed a similar obesity pattern, but the “responder receivers” (RR) displayed significantly different circulating levels (*i.e*., higher) of fasting glucose and leptin and increased homeostasis model assessment (HOMA) values. In contrast, the plasma concentrations of triglycerides, cholesterol and high density lipoproteins (HDL) were similar in the two groups. Furthermore, the authors used a liver histological analysis to demonstrate that the RR mice developed a more severe steatosis and accumulated more triglycerides compared with those of the “not responder receivers” (NRR) group. Moreover, the authors analyzed the expression of the genes involved in lipid uptake, lipogenesis, and FA catabolism, and the expression of the enzymes involved in *de novo* lipogenesis was increased in the RR group compared with that in the NRR group; similarly, the expression increased for CD36, a molecule that imports different lipids and lipoproteins. The authors did not find a significant difference in the systemic and hepatic inflammation status in the two groups. No significant differences in the plasma concentrations of pro-inflammatory cytokines, TNFα, IL-1β, IL-6, IL-10, transforming growth factor (TGF) β and TLRs were found. These data suggest that the gut microbiota affects the host metabolism independently of the immune system. In contrast, the gut microbiota differed between the RR and NRR mice. Differently from NRR mice, the RR mice showed later hepatic macrovesicular steatosis, confirmed by a higher content of triglycerides in the liver and by an increased expression of genes engaged in *de novo* lipogenesis [99].

Yin *et al*. [103] assayed the gut microbiota modulation in an HFD-induced NAFLD rat model. A Chinese herbal formula (CHF), a remedy that is generally used to reduce body weight, lighten HS, and decrease the triglyceride and FFA content, was administered to these rats. The gut microbiota from the CHF-treated and the control rats were analyzed by PCR-DGGE and 16S rRNA V3 region pyrosequencing. Both analyses indicated a significantly difference in the gut microbiota of the two groups. In particular, the *Escherichia*/*Shigella* genera were significantly more abundant in the HFD-fed rats than in the controls but decreased to the control levels after the CHF treatment. The genus *Collinsella* (*i.e*., Actinobacteria), a known producer of SCFA, was significantly elevated in the CHF-treated rats compared with the HFD-fed rats [103].

De Minicis *et al*. [104] subjected control- and HFD-fed mice to either bile duct ligation (BDL) or hepatotoxin carbon tetrachloride (CCl_4_) injections. Previously gut-sterilized mice were subjected to microbiota transplantation by oral gavage of the cecum content obtained from control- or HFD-fed donors. The fibrosis, intestinal permeability, bacterial translocation and serum endotoxemia were measured. The inflammasome components were evaluated in the gut and in the liver. The assumed dysbiosis of the microbiota was evaluated by pyrosequencing. The degree of fibrosis was increased in the HFD + BDL mice compared with the control + BDL mice, while no differences were observed between the control + CCl_4_ mice and the HFD + CCl_4_ mice. Cultures of the mesenteric lymph nodes showed a higher density of infection in the HFD + BDL mice than in the control + BDL mice, suggesting a higher bacterial translocation rate. Metagenomics revealed a reduced ratio between Bacteroidetes and Firmicutes with a dramatic increase of Proteobacteria in the HFD + BDL mice compared with the control + BDL mice. The inflammasome expression was increased in the liver of the fibrotic mice but was significantly reduced in the gut. Furthermore, the microbiota transplantation revealed more liver damage in the control diet-fed mice than in the HFD-treated mice that also received a microbiota transplant; the liver damage was enhanced by transplantation of selected Gram-negative bacteria, which were obtained from the cecum content of HFD + BDL-treated mice. Therefore, dietary habits, by increasing the percentage of Gram-negative intestinal endotoxin producers, may accelerate liver fibrogenesis, thus introducing dysbiosis as a co-factor that contributes to chronic liver injury in NAFLD [104].

Additionally, Sawada *et al*. [109] investigated the role of palmitic acid (PA) in triggering the development of a pro-inflammatory state of NAFLD in the mouse model. In this work, NAFLD was induced in HFD-fed mice. The mice were sacrificed, and the expression of TLRs, TNF, IL-1β, and phospho-IL-1 receptor-associated kinase in the liver and small intestine were assessed. Additionally, hepatocytes and KCs were treated with PA to evaluate its effects on TLR induction. The results showed that the expression of inflammatory cytokines, such as TNF, IL-1β, and TLR-2, TLR-4, TLR-5, and TLR-9, was increased in the liver but was decreased in the small intestine of the HFD-fed mice, while the expression of TLRs in the primary hepatocytes and KCs was increased by treatment with PA. Therefore, in the development of the NAFLD pro-inflammatory state, PA may trigger the expression of TLRs, which contribute to the induction of inflammatory cytokines via TLR signals through intestinal microbiota [109].

Generally, murine models have provided evidence that inflammasome-deficiency-associated changes in the configuration of the gut microbiota strongly correlate with a more severe steatosis and inflammatory state, leading to the progression to NASH in the presence of TLR-4 and TLR-9 agonists. Co-housing wild-type mice with inflammasome-deficient mice may induce hepatic steatosis and obesity exacerbation, suggesting that the interactions between the gut microbiota and the host are crucial for metabolic homeostasis maintenance and highlighting the pivotal role of the gut microbiota in the pathogenesis of apparently hepato-metabolic diseases, such as NAFLD [94]. Gut microbiota may also affect the lipid metabolism regulation of the host. Pachikian and coworkers [105] have recently shown that feeding mice a diet depleted in n-3 polyunsaturated FA (PUFA) results in hepatic alterations similar to those observed in NAFLD patients. Using this mouse model, they reported that fructo-oligosaccharide (FOS) supplementation modifies the gut microbiota, reduces cholesterolemia and reverses the hepatic lipid accumulation in n-3 PUFA-depleted mice. In particular, C57BL/6J mice fed an n-3 PUFA-depleted diet and treated for 3 months with FOS displayed a higher cecal content of *Bifidobacterium* spp., a lower content of *Roseburia* spp. and reduced hepatic triglyceride accumulation compared with those in the control group. Therefore, specific nutrients may provide a potentially beneficial treatment for NAFLD by modulating the gut microbiota [105,109].

### 4.2. The Present Knowledge about NAFLD Patients

The number of descriptive studies on gut microbiota composition under NASH and NAFLD conditions is still insufficient to fully elucidate the “type” and role of gut microbes in liver damage. Because NAFLD is considered to be a multifactorial disorder that arises from genetic, environmental, metabolic and inflammatory contributions, the link between the gut microbiota, host-commensal metabolism and liver function must be thoroughly explored by investigating the main metabolome pathways and components. It is believed that many factors in addition to fatty deposition, such as reactive oxygen species, TLR signaling, signals from adipose tissue (e.g., Fas and cytokines), diet, genetic factors and the immune system, may contribute to the progression along the NAFLD spectrum to NASH [3,110]. Despite its importance, the process driving NAFLD to NASH is not fully understood. Among all the factors, diet plays a crucial role not only in fat deposition but also in the possible interactions with the gut microbiota. Indeed, some preliminary research studies in the obesity field have revealed a possible connection between diet, microbiota structure modulation and NASH development [111]. Despite significant inter-individual variations in the gut microbiota composition, the majority of healthy intestinal microbiota comprises Bacteroidetes and Firmicutes as the predominant phyla [53]. Their ratio has been found to be altered under obesity conditions in mice models and in humans [100,111,112]. The bioreactor microbiota generates many metabolic products, including ethanol and other compounds that may have toxic effects on the human host after intestinal absorption and then transfer to the liver. A recent paper suggested that the composition and the *in silico* inferred metabolic network of the gut microbiota in obese humans is different from that of healthy-weight individuals, suggesting possible metabotypes associated with physiological and diseased microbiota profiles [113].

Raman *et al*. [106] recently compared gut microbiota phylotypes and metabolic profiling (metabolome) in a group of 30 obese NAFLD patients with an age-matched control for every patient; the biochemical and metabolic parameters were well characterized for these patients. Fecal microbiota OTUs were characterized by a multitag pyrosequencing-based NGS, and volatile organic compounds (VOC) profiles were measured by GC-MS. The NGS of the fecal microbiome in the NAFLD patients revealed a statistically significant over-representation of *Lactobacillus* spp. (family Lactobacillaceae), *Dorea* spp., *Robinsoniella* spp., and *Roseburia* spp. (family Lachnospiraceae); both families belong to the phylum Firmicutes. However, one member of the phylum Firmicutes was significantly under-represented in the fecal microbiome of the NAFLD patients (*Oscillibacter* spp., family Ruminococcaceae) (Table 1). The fecal VOC profiles from the obese NAFLD and healthy patients differed, and a significant increase in ester compounds in the NAFLD patients was associated with the compositional shifts in the microbiome. In addition, a recent cross-sectional study [100] aimed to identify the differences in gut microbiota between adults with biopsy-proven NAFLD (e.g., categorized as simple steatosis, SS or NASH) and living liver donors as healthy controls (HC). Fifty subjects were recruited; of these subjects, 11 had SS, 22 had NASH, and 17 had HC. A stool sample was collected from each patient along with clinical and laboratory data, food records, and physical activity logs. Quantitative real-time PCR was used to measure the total bacterial counts of Bacteroidetes (e.g., *Bacteroides*/*Prevotella*), Actinobacteria (e.g., family Bifidobacteria, species *Clostridium leptum*, *Clostridium coccoides*), and Proteobacteria (e.g., *Escherichia coli*). The NASH subjects displayed clinical phenotypes with higher levels of transaminase (alanine transaminase or ALT, aspartate aminotransferase or AST, and HOMA) indexes for insulin resistance and beta-cell function but comparable values of alkaline phosphatase (ALP), glucose, hemoglobin A1c, cholesterol, and triglyceride levels. Furthermore, 80% of the NASH patients showed a variable degree of fibrosis (ranging from F1–F4). The patients with NASH showed a lower percentage of Bacteroidetes compared with both SS and HC patients and higher levels of *C. coccoides* compared with SS patients. No differences were observed for the remaining bacteria. However, the BMI and dietary fat intake differed between the groups; therefore, the authors performed a linear regression that adjusted for these variables. This analysis indicated that the differences in *C. coccoides* concentration were no longer significant, while the lower percentage of Bacteroidetes continued to be significantly associated with the presence of NASH, suggesting an inverse and diet-/BMI-independent association between NASH and the Bacteroidetes percentage in the gut microbiota [100] (Table 1).

In pediatric subjects, Zhu and co-workers [108] used 16S rRNA-based pyrosequencing to determine for the first time the composition of gut microbiota in NASH, obese, and healthy children. Additionally, the ethanol blood levels were measured to monitor the total amount of endogenous ethanol in both the patients and the healthy controls using an ethanol assay based on alcohol oxidase catalysis and colorimetric detection. Under normal conditions, ethanol is constantly produced in the human body, and the gut microbiota is the major source of endogenous alcohol, as suggested by the increased blood alcohol level after the intake of alcohol-free food [114]. The ethanol is immediately and almost completely removed from the portal venous system by liver alcohol dehydrogenases (ADHs), catalases, and the microsomal ethanol-oxidizing system (MEOS). Indeed, when ADH is inhibited, the blood alcohol levels increase [114]. The production of ethanol in the gut is also supported by the fact that the liver and GI tract have the highest ADH activities [115]. UniFrac-based analysis indicated that the sequences were clustered by disease phenotype. Based on this result, each generated set was associated with a distinctive enterotype. Differences between healthy and obese subjects (with or without NASH) were abundant at each taxon level while fewer differences were observed between the obese and NASH microbiota components. In detail, Proteobacteria, Enterobacteriaceae, and *Escherichia* spp. were the only phylum, family and genus OTUs showing significant differences (>1%) between the obese and NASH microbiomes. Remarkably, comparable levels of blood ethanol were observed between the healthy subjects and the obese non-NASH patients, while the NASH patients exhibited significantly elevated blood ethanol levels (Table 1). Zhu and co-authors [108] classified the NASH-related microbioma samples into three enterotypes using the criteria previously described by Arumugam *et al.* [52]: 53/63 samples fit into enterotypes 1 (rich in *Bacteroides*), 2 (rich in *Prevotella*) and 3 (with low levels of both *Bacteroides* and *Prevotella*), while the remaining samples (10/63), which were abundant in both *Bacteroides* and *Prevotella*, were grouped into a hybrid enterotype. The analyses revealed that *Prevotella* was poorly represented in the individuals, who were usually classified as enterotype 1 or 3, in contrast to the obese and NASH patients, who were frequently classified as enterotype 2. At the phylum level, the authors found that Bacteroides and Firmicutes were the dominant phyla in the NASH patients. Two other phyla (Actinobacteria and Proteobacteria) were also present and showed >1% abundance in at least one of the groups. Finally, an increase in Bacteroidetes and a decrease in Firmicutes were observed in both the obese and NASH groups compared with normal-weight individuals, but the difference was not statistically significant. Moreover, the phylum Actinobacteria was found to be lower in the NASH group than in the healthy subjects, but the only phylum whose abundance significantly differed was Proteobacteria. In detail, in the Actinobacteria phylum, the authors [108] observed a progressive decrease in the abundance of Bifidobacteriaceae and *Bifidobacterium* spp. in the healthy group compared with the NASH group. In contrast, within the phylum Bacteroidetes, the family Prevotellaceae was much higher in the obese and NASH samples than in the controls. Most of the Prevotellaceae sequences belonged to the genus *Prevotella* spp. Within Firmicutes, the families Lachnospiraceae and Ruminococcaceae were identified by Zhu *et al*. [108] as the main cause of the observed decrease to a similar extent in both the obese and NASH groups. Furthermore, the most abundant genera in the Firmicutes phylum, *Blautia* and *Faecalibacterium*, showed a large reduction in abundance in the obese and NASH groups, while the observed increase of Proteobacteria in the obese and NASH individuals was explained by the elevated abundance of the Enterobacteriaceae family. Most of the Enterobacteriaceae sequences belonged to *Escherichia* spp., which is the only abundant genus showing a significant difference between the obese and NASH groups. This finding is remarkable because *Escherichia* spp. under anaerobic conditions, such as many other Enterobacteriaceae genera, is able to produce ethanol as a product of a mixed-acid fermentation pathway, suggesting a correlation between the presence of alcohol-producing bacteria and the development of NASH. The increased abundance of alcohol-producing bacteria and the elevated blood-ethanol concentration in NASH patients corroborate the role of alcohol-producing microbiota in the pathogenesis of NASH. The distinct composition and related metabolic activity of the gut microbiome among the NASH, obese, and healthy controls actually seems to suggest a target for intervention or a marker for disease [116].

The only paper on pediatric subjects produced to date [108] reports that the levels of Firmicutes and Actinobacteria are lower than those than in control groups, while the Proteobacteria and Bacteroidetes are over-represented in the NASH group compared with the healthy group. Similar levels were reported for Actinobacteria and Proteobacteria, while differences were observed among the adult cases for Firmicutes and Bacteroidetes. Therefore, a possible Proteobacteria-like enterotype could be inferred; this enterotype mainly extracts energy from carbohydrates and produces lactate, acetate, succinate, and formate as the main fermentation products [117]. Proteobacteria include the bacterial families Enterobacteriaceae (*i.e*., *E. coli*), Oxalobacteriaceae, Pseudomonadaceae, Desulfovibrionaceae, and Helicobacteraceae, and many of these families are associated with pathogenic species. Remarkably, a vegetarian diet is known to affect the abundance of *E. coli* [91]; thus, this type of diet may be a potential intervention tool to equilibrate the Proteobacteria prevalence. However, there are only a few literature reviews about this topic, and the number of selected cohorts that can be used to infer the possible phylotype and metabolite signatures that correspond to NAFLD/NASH is still limited. Therefore, further descriptive and integrated omics and meta-omics studies are now particularly crucial for highlighting the metabolic nodes of the gut and liver and for providing clinical insight into this disease.

### 4.3. Gut-Induced Modulation: The Role of Prebiotics and Probiotics as External Xenobiotic Stimuli

Over the past few years, novel preventive and/or therapeutic strategies for NAFLD patients have been focusing on the use of prebiotics, probiotics and antibiotics to modulate the gut microbiota, reduce intestinal permeability, increase SCFA production and gut hormones and enhance insulin sensitivity [36,40,105,118]. However, these beneficial effects on the gut microbiota require further investigation because the effects have only been demonstrated in animal models and in limited human studies. Recently, Wong *et al*. [107] characterized the gut microbiota of histology-proven NASH patients by performing ribosomal pyrosequencing. In detail, 20 NASH patients were randomized to receive a mixed probiotic-prebiotic treatment (Lepicol probiotic and prebiotic formula) or the usual care. At baseline and after six months, all patients underwent proton-magnetic resonance spectroscopy (^1^H-MRS) to measure their intra-hepatic triglyceride content. The pyrosequencing did not reveal significant changes; indeed, Bacteroidetes was the most abundant phylum in both groups, while the abundance of Firmicutes was significantly lowered in the NASH patients. The order Aeromonadales, the families Succinivibrionaceae and Porphyromonadaceae, and the genera *Parabacteroides* and *Allisonella* registered a significant increase in NASH patients compared with the controls (Table 1). At month six, fecal analyses were repeated for a longitudinal study [119]. Remarkably, the latter authors found that changes in the fecal microbiota composition positively correlated with the improvement of hepatic steatosis. The reduction in intrahepatic triglyceride content in the NASH patients was actually linked to a reduction of Firmicutes and an enhancement in Bacteroidetes [120,121]. Spencer *et al*. [98] used pyrosequencing to characterize the microbiota of 15 subjects treated with a controlled low choline diet. The authors found a direct correlation between the levels of Gammaproteobacteria and Erysipelotrichi and the changes in liver fat in each subject during the choline depletion phase of the study, while the presence of SNPs at the level of the phosphatidylethanolamine *N*-methyltransferase affected the OTU/liver fat relationship [98]. Various experimental studies and clinical trials have recently revealed the promising effects of probiotics in improving the NAFLD status; however, given the limited experience in this field, the generalization to using probiotics to treat NAFLD requires substantiation through further trials with larger sample sizes and a longer-term follow up [122].

## 5. Conclusions and Future Perspective

Taken together, the “omics” can be used to study the effects of the gut microbiota on the whole host metabolism, resulting in the definition of new metabolic profiles (e.g., -omic charts). This new pathway, the so-called “integrated-omics,” will be able to link the genome features of the gut microbiota to disease phenotypes and thus actively correlate both aspects with the host and microbial metabolic frameworks (http://www.microme.eu/) through the generation and interpretation of large related data sets [123,124].

Once the significance of the gut microbiota metabolism within human metabolic pathways is assessed, “-omics” and meta-omics analyses will enable the identification of an imbalance in diet-induced gut microbiota products, such as LPS, Gram-negative pro-inflammatory molecules, lipids, and glucose; these compounds have already been described as discrete triggers of metabolic diseases but have not yet been located within the interactive metabolic pathways associated with gut microbial networks. These factors are surely active players that drive the progression of NAFLD towards NASH. However, further studies are required to reveal how specific gut microbiota patterns may influence the onset of disease and/or liver derangement through inflammation and fibrosis. Furthermore, the identification of conserved microbiomes in NASH patients (e.g., combined metagenomic and culturomic fingerprinting) might provide an opportunity to design novel and effective preventive and/or therapeutic strategies; direct interventions for the disease may also be possible through the application of systems biology principles to the new era of systems medicine.

## Figures and Tables

**Table 1.** Gut microbiota modifications under NAFLD and specific-diet induced factors compared to healthy controls.

Animal models: induced disease and ameliorating factors										
Disease	Model	Induced	Controls	Age range	Technology and experimental pipeline	Main bacterial phyla tendency				References
						Firmicutes	Proteobacteria	Bacteroidetes	Actinobacteria	
**FL**	Mouse	AF + HFD fed	isocaloric maltose dextrin HFD fed	8–10 week old	V3–V5 16S rRNA pyrosequencing	↓ Lachnospiraceae, Ruminococcaceae; ↑ *Aerococcus* spp., *Listeria* spp., Clostridiales spp., *Allobaculum* spp., *Lactobacillus* spp.	↑ (particularly *Alcaligenes* spp.)	↓ *Bacteroides* spp., *Parabacteroides* spp., *Tannerella* spp., *Halella* spp.	↑ (particularly *Corynebacterium* spp.)	[102]
		AF + LGG	AF			↑	↓	↓	↓	
**NAFLD**	Mouse	RR 3 weeks HFD fed	NRR 3 weeks HFD fed	8 week old	V3–V4 16S rRNA pyrosequencing	↑	Stable	↓	↓	[99]
**NAFLD**	Rat	HFD fed for 6 weeks	normal chow fed for 6 weeks	Not reported	PCR-DGGE and V3 16S rRNA pyrosequencing	↓ *Allobaculum* spp.;↑ *Coprococcus* spp., *Blautia* spp., *Roseburia* spp.	↑ *Escherichia/Shigella*	↓ *Prevotella* spp., *Bacteroides* spp.	↑	[103]
		HFD + QHF	control-HFD			Stable	Stable	Stable	↑	
**NAFLD**	Mouse	HFD fed	control chow fed	8–12 weeks	qRT-PCR and pyrosequencing	↑	Stable	↓	slight ↑	[104]
		HFD + BDL	Control + BDL			↓	↑	↓	↓	
**NAFLD**	Mouse	DEF fed	HFD fed	9 weeks	PCR-DGGE	↑ *Roseburia* spp.	Not reported	Stable	slight ↓	[105]
		DEF + FOS	DEF fed			↓ *Roseburia* spp.	Not reported	Stable	↑ *Bifidobacterium* spp.	
**Human studies: disease-related factors**										
**Disease**	**Model**	**N. enrolled patients**	**N. healthy controls**	**Age range**	**Technology and experimental pipeline**	**Main bacterial phyla tendency**				**References**
						**Firmicutes**	**Proteobacteria**	**Bacteroidetes**	**Actinobacteria**	
**NAFLD**	Humans	30	30	Adults	16S rRNA pyrosequencing	↑ Lactobacillaceae, Veillonellaceae and Lachnospiraceae; ↓ Ruminococcaceae	↑ Kiloniellaceae and Pasteurellaceae	↓ Porphyromonadaceae	Not reported	[106]
**NASH**	Humans	16	22	18–70 years	16S rRNA pyrosequencing	↓ Clostridia and unclassified Firmicutes	↑ Succinivibrionaceae	↑ Porphyromonadaceae	↓	[107]
**NASH**	Humans	22	16	Children and adolescents	16S rRNA pyrosequencing	↓	↑	↑	↓	[108]
**NASH**	Humans	22 NASH + 11 SS	17	Adults	qPCR	↑ *Clostridium coccoides*	Equivalent presence of *Escherichia coli*	↓	Stable	[100]

FL: fatty liver; AF: alcohol-fed; HFD: high-fat diet; LGG: *Lactobacillus rhamnosus* GG; RR: responder receiver; NRR: not responder receiver; QHF: Qushi Huayu Fang, Chinese herbal formula; BDL: bile duct ligation; DEF: *n-3* PUFA-depleted diet; FOS: fructo-oligosaccharides; qRT-PCR: quantitative real-time reverse-transcription PCR; PCR-DGGE: polymerase chain reaction in denaturing gradient gel electrophoresis; SS: simple steatosis; qPCR: quantitative polymerase chain reaction.
